# Determining the sexual quality of life and related factors in patients referred to the department of cardiac rehabilitation: A cross-sectional study

**DOI:** 10.18502/ijrm.v19i3.8574

**Published:** 2021-03-21

**Authors:** Iman Taghizadeh Firoozjaei, Mohsen Taghadosi, Zohre Sadat

**Affiliations:** ^1^Medical Surgical Nursing Department, Faculty of Nursing and Midwifery, Kashan University of Medical Sciences, Kashan, Iran.; ^2^Trauma Nursing Research Center, Kashan University of Medical Sciences, Kashan, Iran.

**Keywords:** Sexual health, Patients, Cardiovascular rehabilitation.

## Abstract

**Background:**

One of the neglected issues among cardiovascular participants is sexual activity, which can affect their general quality of life.

**Objective:**

To evaluate the sexual quality of life and its related factors.

**Materials and Methods:**

This cross-sectional study was conducted on 200 cardiovascular participants, referred to the department of rehabilitation of the Shahid Beheshti Hospital of Kashan, Iran in May 2017. Individuals who met the inclusion criteria filled the demographic characteristics questionnaires, including sexual quality of life questionnaire (SQOL)-female and SQOL-male, sexual knowledge post-myocardial infarction scale (SKS-MI), sexual knowledge after coronary artery bypass grafting surgery scale (SKS-CABG), and couple communication scale (CCS).

**Results:**

In this research, the mean score of SQOL of the participants was 50.02 ± 22.57. According to the results, there was a significant and positive association between the scores of SQOL and CCS (r = 0.540, p ≤ 0.0001), SKS-MI (r = 0.322, p = 0.006), and SKS-CABG (r = 0.178, p = 0.046). The maximum association was observed between SQOL and CCS and the minimum association between SQOL and SKS-CABG. Moreover, there was a relationship between the SQOL and participant's age and level of education.

**Conclusion:**

According to the results of the study, the sexual quality of life scores among participants with MI and CABG was not favorable and this participant suffered from a lack of sexual knowledge.

## 1. Introduction 

Cardiovascular diseases are one of the main causes of mortality in the world, 80% of deaths occur in middle- and low-income countries (1). Sexual quality of life (SQOL) is one of the main issues in the area of sexual health (2). Similar to the general quality of life, SQOL refers to the individual's perception of their position in the context of the culture, values, goals, expectations, standards, and priorities (3). In cardiovascular patients, there is a high prevalence of sexual dysfunction and low quality of sexual life, which remains unchanged in more than half of the patients despite their improved heart conditions after surgery (4).

In research by Schumann, it was reported that 59.2% of the patients had impaired sexual performance after coronary artery bypass graft (CABG) (5). In another study conducted in Ireland, 48% of the patients were dissatisfied with their sexual activities after CABG (6). Similarly, Pournaghash and colleagues marked that 85.5% of the participants had unfavorable sexual performance after CABG (7). Ivarsson in a study found that sexual dysfunction was significantly common among female and male cardiovascular patients after heart failure (8). Lack of sexual satisfaction can lead to reduced health, lifespan, satisfaction with marital life, disruption of growth, and excellence of couples and unraveled marriage (9).

Bagheri and colleagues expressed in research that heart failure in patients was associated with severe problems in sexual relations, including frequency and duration of relation and fear of recurrence of heart failure. Therefore, the level of sexual activities significantly decreased in these patients (10). However, other study has indicated that fear of death in these patients can result in increased attention, intimacy, mutual understanding, desire to be with each other, as well as increased sexual satisfaction and quality of life (11). Couple communication can improve the quality of sexual life, and increased intimacy between couples can lead to an elevated number of and satisfaction with sexual relations, which eventually leads to improved quality of sexual life (12).

In research by Teles, it was shown that the increased age of individuals was associated with a decreased level of sexual activities (13). Religious teachings have a special place in sexual relations, and cultural norms and economic status are among the effective factors for the quality of sexual life (14). Complications, such as decreased quality of sexual life, have been caused due to an increasing trend of heart disease. In Iran, there is insufficient information on the quality of sexual life and its related factors in this group of patients. In this study, the factors that affect the quality of sexual life of participants in the field of sexual knowledge, which have not been considered in previous studies, have been studied.

Therefore, the present study aimed to evaluate the quality of sexual life and its related factors in patients who were referred to the Department of Rehabilitation at the Shahid Beheshti Hospital of Kashan, Iran in May 2017. Our findings can help nurses, as well as health managers and decision-makers, to take appropriate measures toward sexual health education and improvement of the quality of sexual life of patients by evaluating the sexual needs of these individuals and selecting proper strategies in this regard.

## 2. Materials and Methods

This cross-sectional research was conducted on 200 cardiovascular patients who referred to the Department of Rehabilitation Shahid Beheshti Hospital of Kashan in may 2017. The sample size was estimated by performing a pilot research on 20 eligible individuals, with a mean and standard deviation of a score of sexual health quality calculated at 50.20 ± 22.1. In total, 178 subjects were selected using the equation below: 

$$n=\frac{z_{1-\frac{\alpha }{2}}^{2}\times \delta^{2}}{d^{2}}=\frac{\left({1/96}^{2}\right)\times \left({22/1}^{2}\right)}{{\left(0/15\right)}^{2}}=178$$

$$z_{1-\frac{\alpha }{2}}=\left(1/96\right)$$

$$\delta^{2}=\left({22/1}^{2}\right)$$

Based on the standard deviation of SQOL in Dr. Sadat's study (15). 

$$d^{2}={\left(0/15\right)}^{2}$$

However, the study was performed on 200 individuals for higher accuracy. The inclusion criteria were: an age range of 20-65 yr, married, suffering from cardiovascular problems (myocardial infarction during the past two-five months, undergoing CABG in the past one-four months) which was detected by a cardiologist, lack of detected mental problems, literate, and lack of history of medication use due to sexual problems and prohibition of sexual health by the order of a cardiologist after the start of cardiovascular problems. The exclusion criteria, on the other hand, were incomplete filling of the questionnaire and the reluctance to continue to participate in the research.

### Research tools

While all of the subjects filled the demographic characteristics questionnaire (age, gender, place of residence, occupation, smoking status, monthly income, number of children, level of education, blood pressure level, diabetes) and the couple communication scale (CCS), only women completed the SQOL-female (SQOL-F) and just men filled the SQOL-male (SQOL-M). In addition, the participants completed one of the sexual knowledge post myocardial infarction scale (SKS-MI) and sexual knowledge post CABG (SKS-CABG) according to their type of disease.

### SQOL

The 11-item SQOL-M questionnaire was developed by Abraham. It is scored based on the Likert scale from completely agree (1) to disagree (6). The maximum and minimum scores of the questionnaire were 66 and 11, respectively. The 18-item SQOL-F was developed by Symond. It is scored based on a six-point Likert scale, where the minimum and maximum scores are 18 and 108, respectively. The reliability and validity of the SQOL-M has been confirmed in Iran and the Cronbach's alpha was 0.82 (15).

In the present study, the reliability of the tool was evaluated by a test-retest on 20 eligible individuals, which was estimated at the Cronbach's alpha of 0.85. The reliability and validity of the SQOL-F questionnaire has also been evaluated and confirmed in Iran and estimated at the Cronbach's alpha of 0.78. Before using the tool in the present study, its reliability was calculated at the Cronbach's alpha of 0.78 using test-retest reliability (16). In addition, for homogenizing scores of SQOL-M and SQOL-F questionnaires, the scores transformed to ranging from 0 to 100.

### CCS

This scale was used in research by Chung on patients after CABG and its Cronbach's alpha was reported as 0.97. This 11-item tool is scored from never (0) to always (3), where the minimum and maximum scores are 0 and 33, respectively. The higher scores are indicative of better couple communication (4). This tool has not been psychometrically evaluated in Iran. Therefore, after the translation of the questionnaire, its content validity was evaluated by 10 experts in a standard way. In addition, test-retest was used to determine the reliability of the questionnaire by distributing the tool among 20 eligible individuals and recompletion of the questionnaires by the same people 10 days later. In the end, the Cronbach's alpha was estimated at 0.89 (4).

### SKS-CABG

This scale was applied by Chung in research on cardiovascular patients after CABG. This 20-item tool evaluates the level of sexual knowledge of patients after CABG, and each of its items is scored 1 (true) and 0 (false) (4). The score range of the questionnaire is 0-20. In the research by Chung, the Cronbach's alpha of the questionnaire was estimated at 0.73. After translating the tool by a standard tool, the questionnaire was distributed among 10 experts in the field of cardiovascular diseases and sexual issues. After their evaluations, experts eliminated the items of 18-20 from the questionnaire for cultural adaptation. The reliability of the tool was determined using test-retest, where Cronbach's alpha was estimated at 0.72 (4).

### SKS-MI

Stink and Swan used 25 items of sexual knowledge-related myocardial infarction patients in the SKS in their study on patients with a history of myocardial infarction (17). This 25-item scale is scored 3 (yes), 2 (no opinion), and 1 (no) and the score range is 25-75. The higher score is indicative of a higher level of sexual knowledge of individuals. To determine the validity and reliability of the scale in Iran, the translation was performed in a standard technique, and then to determine content validity, the Persian version of the questionnaire was provided to 10 experts in the area of cardiovascular diseases and sexual issues. After the assessment, experts eliminated the items of 8, 17, and 19 from the questionnaire for cultural adaptation. Therefore, the final Persian version had 21 items, where individuals received scores within the range of 21-63. Moreover, the test-retest reliability was applied to determine the reliability of the scale, for which the Cronbach's alpha of 0.70 was calculated.

### Ethical considerations

In order to adhere to the ethical considerations, the study was initiated after receiving approvals from the ethics committee (IR.KAUMS.NUHEPM.REC.1396.8) of the Kashan University of Medical Sciences and the necessary permissions from the authorities of the university along with an introduction letter from the research deputy of the aforementioned university. Moreover, the objectives of the research were explained and written informed consents were obtained from the participants prior to the study.

Subjects were also ensured of the confidentiality terms regarding their personal information. Furthermore, participation in the research was voluntary and subjects were able to withdraw from the study at any time. While the questionnaires were filled in the form of self-report, trained nurses (same gender as patients) were used to interview the illiterate subjects and obtain their information. All of the participants filled the questionnaires in a private and quiet place in the Beheshti Hospital of Kashan city.

### Statistical analysis

Data were analyzed by the SPSS software (Statistical Package for the Social Sciences, version 16.0, SPSS Inc., Chicago, Illinois, USA) using descriptive statistics to evaluate and describe the demographic and clinical characteristics. Furthermore, the factors related to the SQOL were assessed using analytical statistics. Before performing the data analysis, the Kolmogorov-Smirnov test was used to evaluate the normality of the variables. According to the results, while two variables of SQOL and SKS-MI had normal distributions (sig > 0.05), the variable of SKS-CABG and CCS had abnormal distribution (sig < 0.05), also demographic characteristics had normal distributions (sig > 0.05). Therefore, the relationship between SQOL and demographic characteristics was assessed using a *t* test and analysis of variance. In addition, the Pearson's correlation coefficient was applied to evaluate the correlation between quantitative variables with normal distribution and Spearman's correlation coefficient to assess the correlation between variables, which had abnormal distribution. The confidence level in this study was considered below p = 0.05.

## 3. Results

In this research, the mean score of SQOL was 50.02 ± 22.57, with mean scores of 50.70 ± 19.53 and 49.60 ± 24.30 for female and male participants, respectively. The confidence level in this study was considered below p = 0.05. While the mean score of female subjects was higher, compared to male participants, results demonstrated no significant difference in this regard (p = 0.928). According to the results, the mean score of SKS-MI was 49.85 ± 5.06 in women and 46.31 ± 4.74 in men, where a significant difference was observed between the participants (p = 0.017). On the other hand, the mean score of SKS-CABG was higher in women with a mean of 9.95 ± 2.33 compared to men with a mean of 9.66 ± 1.77. In terms of CCS, the mean scores of 17.14 ± 8.71 and 18.24 ± 8.63 were obtained for women and men, respectively. However, the difference between the scores was not significant (Table I).

Among the demographic characteristics, a significant relationship was observed between literacy (p = 0.003) and SQOL, in a way that increased level of literacy was associated with improved SQOL. Therefore, individuals with academic education had a higher score of sexual life quality compared to those with a high school diploma. Another significant relationship was observed between age (p = 0.001) and sexual life quality, meaning that the aging of individuals led to decreased scores of SQOL. In addition, a significant association was found between diabetes (p ≤ 0.0001) and sexual life quality, in a way that diabetics had a lower SQOL score, compared to healthy individuals. Other variables (including occupational status, place of residence, income level, housing status, blood pressure level, smoking status, and a number of children) had no significant relationship with the scores of SQOL. According to these results, no significant relationship was observed between the underlying diseases (e.g., hypertension) and sexual life quality. However, its level was higher in subjects without hypertension compared to those with hypertension. While smoking had no significant relationship with sexual life quality, there were lower scores of SQOL in those who smoked compared to those who did not (Table II).

Results demonstrated a significant and positive association between the scores of SQOL and the scores of SKS-MI (r = 0.322, p = 0.006), the scores of SKS-CABG (r = 0.178, p = 0.046), as well as the scores of CCS (r = 0.540, p ≤ 0.0001), where higher scores are indicative of improved sexual life quality (Table III).

**Table 1:** Associations between scores of SQOL, CCS, SKS-MI, and SKS-CABG**${}^{\#}$** with gender


**Variables**		**N**	**Mean ± SD**	**P-value**
**SQOL**				
	**Male**	124	49.60 ± 24.30	0.928
	**Female**	76	50.70 ± 19.53	
**CCS**				
	**Male**	124	18.24 ± 8.63	0.407
	**Female**	76	17.7 ± 8.71	
**SKS-MI-**				
	**Male**	44	46.31 ± 4.74	0.017
	**Female**	28	49.85 ± 5.06	
**SKS-CABG**				
	**Male**	80	9.66 ± 1.77	0.470
	**Female**	48	9.95 ± 2.33	
${}^{\#}$Sexual quality of life, sexual knowledge after coronary artery bypass grafting surgery scale, sexual knowledge post-myocardial infarction scale, couple communication scale *t* test				

**Table 2:** Demographic and clinical characteristics of participants (related factors) and SQOL scores of participants (related factors) and SQOL scores


	**N (%)**	**Mean ± SD**	**P-value**
**Age (yr)**				
	**20–50**	72 (36)	57.22 ± 21.11	0.001**
	**51–59**	102 (51)	47.58 ± 23.22	
	**60–65**	26 (13)	39.60 ± 17.96	
**Gender**				
	**Male**	124 (62)	50.70 ± 19.53	0.739*
	**Female**	76 (38)	49.60 ± 24.31	
**Level of education**				
	**Elementary and junior high school**	111 (55.5)	45.42 ± 23.40	0.003**
	**Diploma**	68 (34)	54.51 ± 19.88	
	**Academic**	21 (10.5)	59.76 ± 21.09	
**Place of residence**				
	**City**	164 (82)	50.82 ± 21.19	0.281*
	**Village**	36 (18)	46.34 ± 28.05	
**Occupational status**				
	**Employee**	31 (15.5)	54.22 ± 22.92	0.373**
	**Self-employed**	86 (43)	48.84 ± 24.81	
	**Housewife**	60 (30)	52.03 ± 19.78	
	**Farmer**	7 (3.5)	38.18 ± 21.69	
	**Retired**	16 (8)	45.76 ± 18.57	
**Income level**				
	**Sufficient**	54 (27)	49.45 ± 21.90	0.977**
	**Medium**	119 (59.5)	50.20 ± 22.41	
	**Insufficient**	27 (13.5)	50.34 ± 25.29	
**Housing status**				
	**Personal property**	144 (72)	51.21 ± 21.77	0.299**
	**Leased**	56 (28)	46.93 ± 24.43	
**Blood pressure**				
	**Yes**	88 (44)	49.89 ± 22.27	0.946*
	**No**	112 (56)	50.11 ± 22.90	
**Diabetes**				
	**Yes**	76 (38)	41.64 ± 23.42	0.000*
	**No**	124 (62)	55.15 ± 20.48	
**Number of children**				
	**1**	145 (66.5)	52.65 ± 21.55	0.64*
	**2**	55 (33.5)	46.16 ± 22.10	
**Smoking status**				
	**Yes**	170 (85)	46.74 ± 20.34	0.389*
	**No**	30 (15)	50.60 ± 22.94	
Data presented as n (%), data presented as Mean ± SD. *t test, **One-way ANOVA				

**Table 3:** Correlation between the scores of SQOL questionnaire and the scores of SKS-MI, SKS-CABG, and CCS


	SKS-MI${}^{**}$	SKS-CABG${}^{*}$	CCS${}^{*}$
**SQOL**	0.322${}^{**}$	0.178${}^{*}$	0.540${}^{*}$
**P-value**	0.006	0.046	0.000
**N**	72	128	200
Pearson${}^{**}$ and Spearman's${}^{*}$ correlations - the sexual quality of life, sexual knowledge after coronary artery bypass grafting surgery scale, sexual knowledge post-myocardial infarction scale, couple communication scale			

## 4. Discussion 

The present study was performed to evaluate the SQOL and its related factors in patients who were referred to the Department of Rehabilitation of the Shahid Beheshti Hospital of Kashan, Iran in 2017. In this research, according to the results, no significant relationship was observed between the scores of SQOL and gender. According to the results of the current research, while there was a positive and significant relationship between SQOL scores and sexual knowledge, and the level of literacy, a significant and reverse association was found between the SQOL scores and age.

In a research conducted by Jaarsma and colleagues a significant relationship was observed between sexual knowledge and decreased fear, which resulted in increased sexual tendency and satisfaction (18). In a previous study by Lai Y-H and colleagues, which was conducted on patients after CABG, a significant association was found between sexual knowledge, couple communication, and age of the patients (4).

In another study, the SQOL of women and men decreased after CABG, in a way that the SQOL score decreased by 50% in the participants who received just half of the complete score. The Moreover, the results of the mentioned study are consistent with our findings, which showed that cardiovascular diseases (e.g., MI and CABG), caused a reduction in SQOL of patients. It is should be mentioned that the score of SQOL in the present study was 50.02 ± 22.57. Studies have shown that decreased levels of SQOL in these patients could be related to the fear of recurrence of cardiac problems, which can result in a decreased tendency to, frequency of, and satisfaction with sexual relations (19, 20). Wrong beliefs (e.g., heart attack or surgery mean reduced or discontinued sexual activity or sexual activity can lead to chest pain (10) and lack of proper knowledge and taboo nature of sexual issues and lack of talking about them with friends and healthcare team can increase the level of fear in patients (21).

According to the results, the mean and standard deviation of the score of SKS-CABG was 9.70 ± 1.99 after CABG. It should be noted that the mentioned score was higher in the research by Chung, changing from 10.69 before the surgery to 13.6 after the surgery (4). This lack of consistency between the results might be due to inadequate education and ignoring the issue of sexual knowledge for cardiovascular patients in hospitals of the country. Therefore, increased education and knowledge of patients can lead to improved sexual life quality, which can consequently enhance public life quality of these people.

According to the results of the present study, a significant correlation was observed between couple communication and sexual life quality (r-0.540). Similar results were obtained by Lai Y-H (4) and colleagues and Sarhadi and colleagues, who mentioned that increased or decreased intimacy between couples led to their increased or decreased level of sexual life (18). Therefore, couple communication can be recognized as a significantly effective factor for the high score of sexual life quality. If a significant correlation between couple communication and sexual life quality is higher than the other factors, that can be attributed to the high impact of the couple's emotional relationship in reducing stress and problems of the patient's partner, reducing her stress and, consequently, increasing their quality of sexual life.

In the present study, the mean score of sexual knowledge post MI was lower, compared to the level of sexual knowledge score after MI in the research by Baron Stom, who used the same questionnaire and reported the score at 51. According to the results of this study, increased sexual knowledge was associated with a higher level of sexual activity, satisfaction, and desire, as well as increased and decreased self-confidence and fear of sexual activity, respectively (22, 23). Therefore, sexual knowledge can be regarded as a determining factor for the quality of sexual life. The level of literacy is another important factor for sexual life quality, in a way that Martin Diaz marked in his research that increased level of education of the participants was associated with a lower level of sexual dysfunction (24). However, Bagheri and colleagues expressed no significant relationship between the level of education and satisfaction with marital relations in patients after MI (25). Our findings also demonstrated a significant association between the level of literacy and sexual life quality, in a way that the level of sexual life quality was higher in individuals with more sexual knowledge compared to those with a lower level of knowledge and literacy.

In research by Greenburg and colleagues (26), a reverse relationship was observed between aging and sexual desire and frequency of sexual relations. In contrast, Bagheri and colleagues found no significant relationship between age and score of marital satisfaction in patients after MI (25). According to the results of the present study, a significant and reverse relationship was observed between age and sexual life quality, in a way that there was a higher sexual knowledge score and sexual life quality in younger adults compared to the elderly. This difference between age groups could be related to reducing sexual desire and strength, which results in a lower tendency to learn about sexual activities and lower literacy levels of individuals (26).

Underlying diseases (e.g., hypertension and diabetes) are other factors that can negatively affect the sexual life quality in both genders. According to the results of the present study, smoking and underlying diseases had negative impacts on sexual life quality. Meanwhile, a significant association was found between diabetes and sexual life quality in both genders (p ≤ 0.0001). This significant difference in male diabetes could lead to erectile dysfunction, which is one of the main factors for sexual relations in men, and when combined with other problems, can lead to decreased sexual life quality.

In female diabetes, problems such as hyperglycemia, infection, neurological and vascular problems, and social-mental issues cause sexual problems, such as pain during the sexual intercourse, dryness, and failure to achieve orgasm in women (26). Other factors, such as the type of analgesic, place of residence, level of income, and a number of children, had no impact on the score of sexual life quality and had no significant relationship with the sexual life quality. Similarly, no significant relationship was observed between these factors and the level of marital satisfaction in the research by Bagheri and colleagues (25).

## 5. Conclusion

The results of the present study showed that the quality of the sexual life of heart patients after SKS- MI andSKS- CABG is not appropriate and these patients suffer from lack of sexual knowledge. This need for patients needs more attention while it is neglected in different societies due to taboo sexual issues, therefore implementing training programs to help increase the sexual knowledge of these patients and thus help to improve the quality of life of these patients is important.

##  Limitations

In the present study, the public questionnaire on the evaluation of sexual life quality was used due to a lack of specialized surveys for assessing sexual life quality in cardiovascular patients. Therefore, it is recommended that specialized tools be used in future studies to more efficiently evaluate the sexual life quality of these patients.

In addition, this research was conducted in one city and on a small sample size. Therefore, performing simultaneous broader studies on larger sample sizes and in various regions of the country can lead to more accurate results on the level of sexual life quality of the patients. Given the apparent need of patients to counseling in the current research, it is suggested that special attention be paid to providing counseling for these patients on the area of sexual activities in future studies. Moreover, nurses and other healthcare team members must receive the necessary educations on sexual counseling.

##  Conflict of Interest

The authors declare no conflict of interest in this study.
