# Corrigendum to “Determining the sexual quality of life and related factors in patients referred to the department of cardiac rehabilitation: A cross-sectional study” [Int J Reprod BioMed 2021; 19: 261-270]

**DOI:** 10.18502/ijrm.v19i5.9259

**Published:** 2021-06-23

**Authors:** Iman Taqizade Firoozjaei, Mohsen Taghadosi, Zohre Sadat

 The publisher has been informed of an error that occurred on page 261 in which the first authors name must be changed to Iman Taghizadeh Firoozjaei. On behalf of the author, the publisher wishes to apologize for this error. The online version of article has been updated on June 16, 2021. 

